# Supplementary material for: Presence, Location and Conservation of Putative G-Quadruplex Forming Sequences in Arboviruses Infecting Humans
Source: Int J Mol Sci. 2023 May 30;24(11):9523. doi: 10.3390/ijms24119523 (PMC10253674; doi:10.3390/ijms24119523)
Supplement: Supplementary file 1 [file ijms-24-09523-s001.zip › ijms-2327452-supplementary figures and tables.pdf]

Supplementary material

Original Research Article

Presence, location and conservation of putative G-quadruplex forming sequences in arboviruses infecting humans

Table S1. Arboviruses PQSs frequency on positive and negative RNA strands. The table reports the analysed viruses in alphabetical order. Columns indicate the virus name (Virus), the viral genomic structure (Genome/Segments) with the name of each segment in case of segmented genomes (Segments), the total number of PQSs predicted in viral genomes (PQSs in viral genomes), the number of PQSs on the positive or negative RNA strand and relative percentages (PQSs on positive/negative strand and % PQSs on positive/negative strand, respectively). Colors corresponds to dsRNA (grey), negative strand RNA (ssRNA-, yellow), positive strand RNA (ssRNA+, blue). In segmented RNA viruses, the viral segments (S, M and L) are ordered by length.

Virus	Genome/Segments	PQSs in viral genomes	PQSs on positive strand	% PQSs on positive strand	PQSs on negative strand	% PQSs on negative strand
Australian bat lyssavirus	Single linear RNA	45	23	51.11	22	48.89
Banna virus	Segment 1	2	1	50	1	50
	Segment 2	4	2	50	2	50
	Segment 3	6	6	100	0	0
	Segment 4	1	1	100	0	0
	Segment 5	2	1	50	1	50
	Segment 6	3	3	100	0	0
	Segment 7	2	1	50	1	50
	Segment 8	1	1	100	0	0
	Segment 9	0	0		0	
	Segment 10	2	1	50	1	50
	Segment 11	3	2	66.67	1	33.33
	Segment 12	2	1	50	1	50
Barmah Forest virus	Single linear RNA	74	26	35.14	48	64.86
Bunyamwera virus	Segment S	4	2	50	2	50
	Segment M	5	3	60	2	40
	Segment L	5	4	80	1	20
Bunyavirus La Crosse	Segment S	4	4	100	0	0
	Segment M	6	4	66.67	2	33.33
	Segment L	8	6	75	2	25
Bunyavirus snowshoe hare	Segment S	3	3	100	0	0
	Segment M	9	7	77.78	2	22.22
	Segment L	5	5	100	0	0
Chandipura virus	Single linear RNA	46	27	58.70	19	41.30

Chikungunya virus	Single linear RNA	65	28	43.08	37	56.92
Crimean-Congo hemorrhagic fever virus	Segment S	6	2	33.33	4	66.67
	Segment M	23	12	52.17	11	47.83
	Segment L	18	17	94.44	1	5.55
Dengue virus 1	Single linear RNA	61	43	70.49	18	29.51
Dengue virus 2	Single linear RNA	64	47	73.44	17	26.56
Dengue virus 3	Single linear RNA	69	55	79.71	14	20.29
Dengue virus 4	Single linear RNA	77	53	68.83	24	31.17
Dhori virus	Segment 1	10	3	30	7	70
	Segment 2	5	3	60	2	40
	Segment 3	7	3	42.86	4	57.14
	Segment 4	11	8	72.73	3	27.27
	Segment 5	4	3	75	1	25
	Segment 6	5	3	60	2	40
Dugbe virus	Segment S	5	5	100	0	0
	Segment M	12	9	75	3	25
	Segment L	18	17	94.44	1	5.56
Eastern equine encephalitis virus	Single linear RNA	59	21	35.59	38	64.41
Isfahan virus	Single linear RNA	33	23	69.70	10	30.30
Japanese encephalitis virus	Single linear RNA	101	71	70.30	30	29.70
Langat virus	Single linear RNA	125	106	84.80	19	15.20
Louping ill virus	Single linear RNA	130	107	82.31	23	17.69
Mayaro virus	Single linear RNA	66	29	43.94	37	56.06
Murray Valley encephalitis virus	Single linear RNA	87	60	68.97	27	31.03
O'nyong-nyong virus	Single linear RNA	53	27	50.94	26	49.06
Oropouche virus	Segment S	4	3	75	1	25
	Segment M	2	1	50	1	50
	Segment L	2	0	0	2	100
Punta Toro phlebovirus	Segment S	4	1	25	3	75
	Segment M	6	6	100	0	0
	Segment L	12	12	100	0	0
Rift Valley fever virus	Segment S	8	2	25	6	75
	Segment M	16	13	81.25	3	18.75
	Segment L	24	16	66.67	8	33.33

Ross River virus	Single linear RNA	76	40	52.63	36	47.37
Sagiyama virus	Single linear RNA	71	31	43.66	40	56.34
Sandfly fever Sicilian virus	Segment S	7	7	100	0	0
	Segment M	17	14	82.35	3	17.65
	Segment L	23	18	78.26	5	21.74
Sandfly fever Toscana virus	Segment S	7	2	28.57	5	71.43
	Segment M	17	10	58.82	7	41.18
	Segment L	21	17	80.95	4	19.05
Semliki Forest virus	Single linear RNA	95	51	53.68	44	46.32
Sindbis virus	Single linear RNA	68	21	30.88	47	69.12
St. Louis encephalitis virus	Single linear RNA	85	66	77.65	19	22.35
Tick-borne encephalitis virus	Single linear RNA	120	95	79.17	25	20.83
Tick-borne powassan virus	Single linear RNA	123	98	79.67	25	20.33
Usutu virus	Single linear RNA	92	62	67.39	30	32.61
Uukuniemi virus	Segment S	8	5	62.50	3	37.5
	Segment M	16	11	68.75	5	31.25
	Segment L	30	17	56.67	13	43.33
Venezuelan equine encephalitis virus	Single linear RNA	63	28	44.44	35	55.56
Vesicular stomatitis virus strain Indiana	Single linear RNA	34	18	52.94	16	47.06
Vesicular stomatitis virus non-Indiana strains	Single linear RNA	29	23	79.31	6	20.69
West Nile virus	Single linear RNA	88	70	79.55	18	20.45
Western equine encephalitis virus	Single linear RNA	55	25	45.45	30	54.54
Yellow fever virus	Single linear RNA	94	73	77.66	21	22.34
Zika virus	Single linear RNA	101	79	78,22	22	21,78

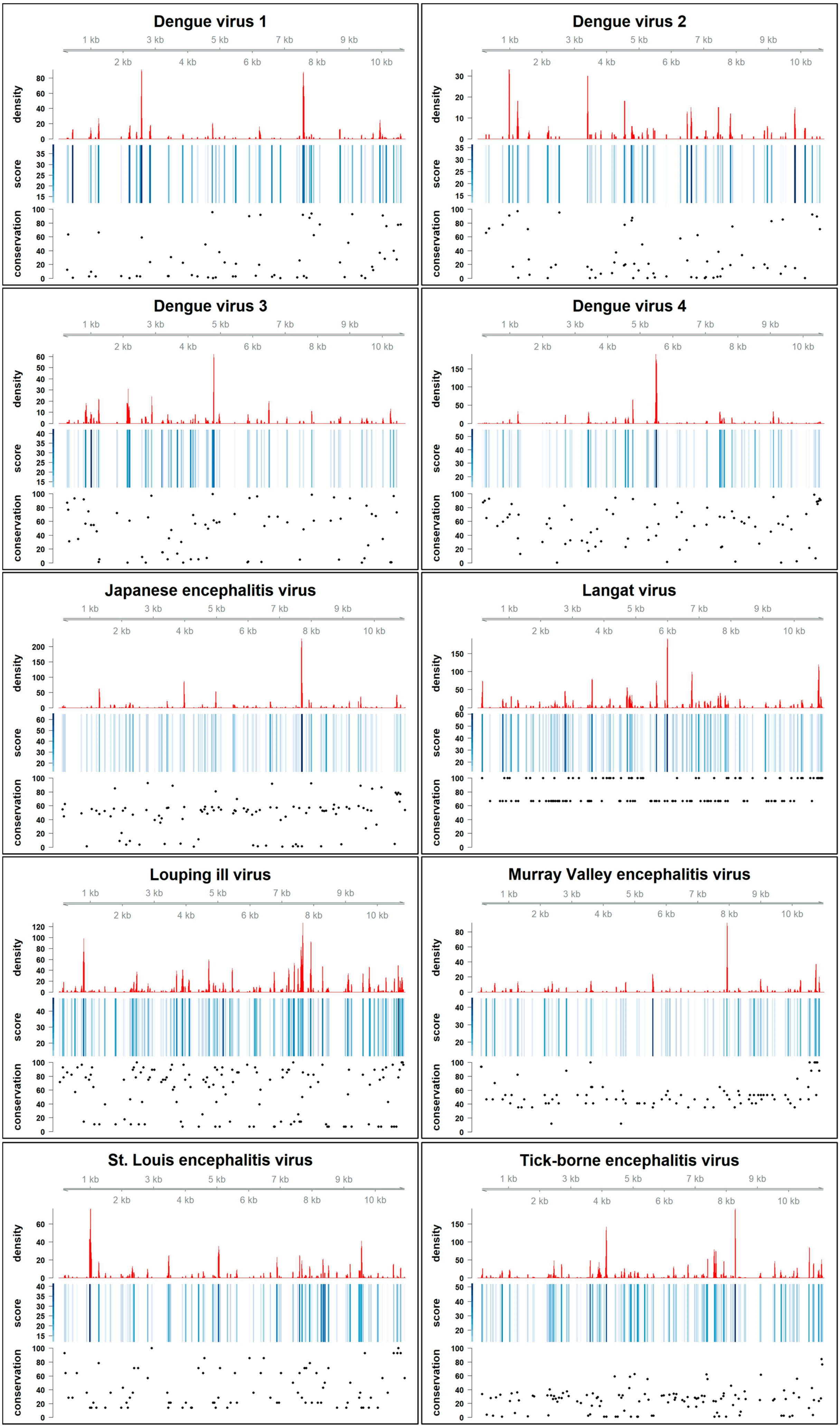
Dengue virus 1
Dengue virus 2
Dengue virus 3
Dengue virus 4
Japanese encephalitis virus
Langat virus
Louping ill virus
Murray Valley encephalitis virus
St. Louis encephalitis virus
Tick-borne encephalitis virus
1 kb
2 kb
3 kb
4 kb
5 kb
6 kb
7 kb
8 kb
9 kb
10 kb
density
score
conservation

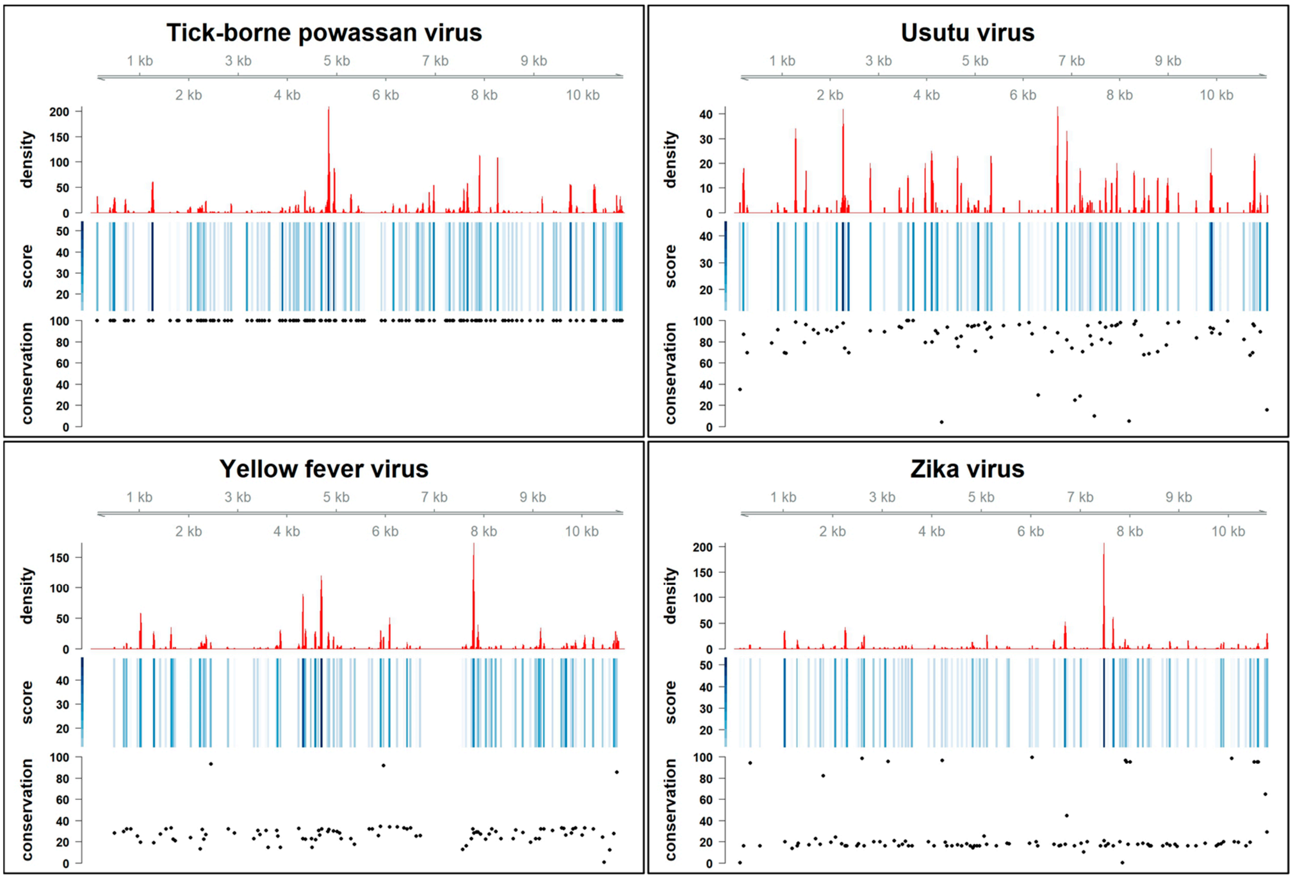

Figure S1. Presence, density, score and conservation of PQSs in positive-strand ssRNA genome viruses (Flaviviruses)

Plots representing the PQS density (red bars), the score (blue bars) and the conservation percentage (black dots) of each predicted PQS. The viral genome length is reported above the density plot.

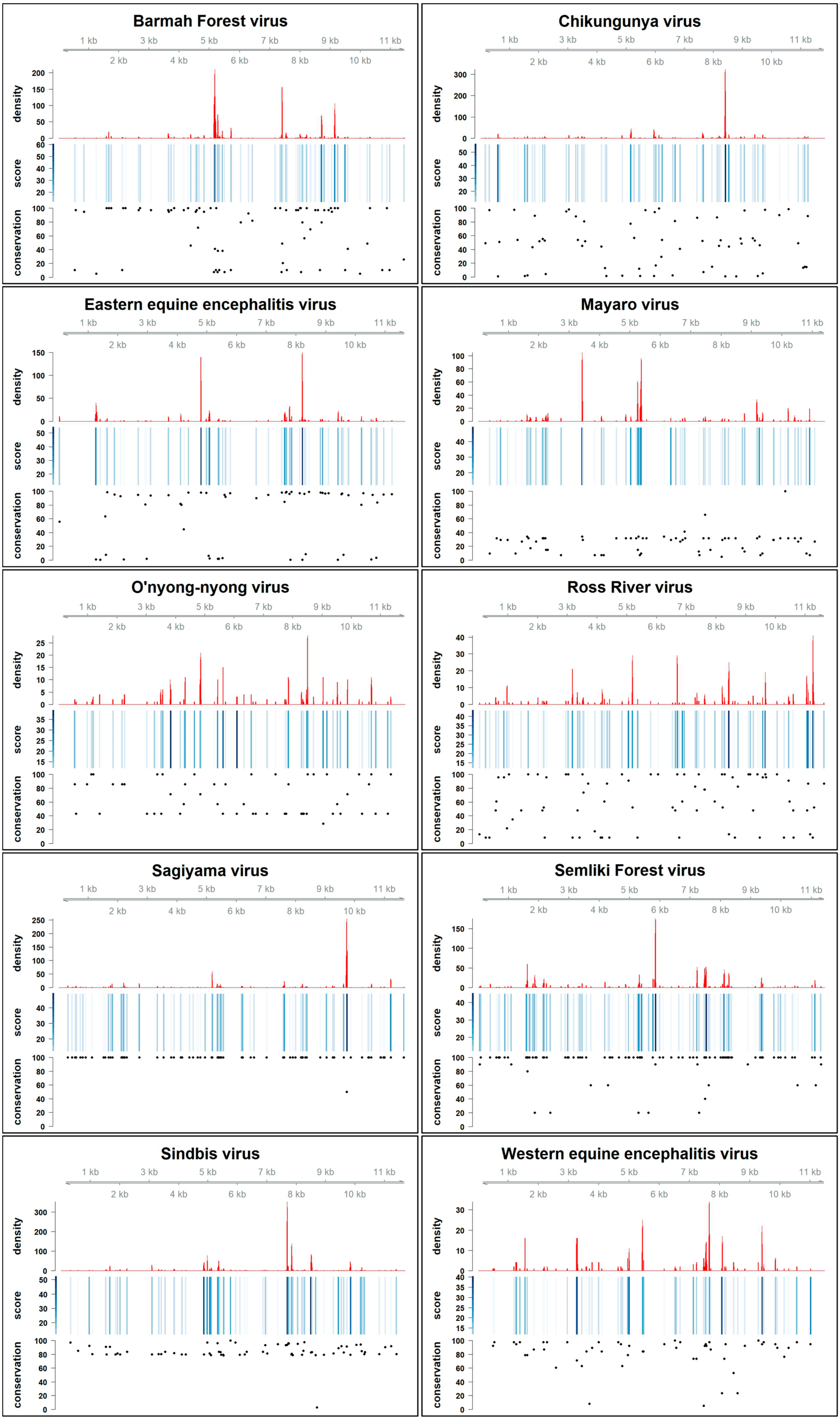
Barmah Forest virus
Chikungunya virus
Eastern equine encephalitis virus
Mayaro virus
O'nyong-nyong virus
Ross River virus
Sagiyama virus
Semliki Forest virus
Sindbis virus
Western equine encephalitis virus
1 kb
2 kb
3 kb
4 kb
5 kb
6 kb
7 kb
8 kb
9 kb
10 kb
11 kb
density
score
conservation

Figure S2. Presence, density, score and conservation of PQSs in positive-strand ssRNA genome viruses (Alphaviruses)

Plots representing the PQS density (red bars), the score (blue bars) and the conservation percentage (black dots) of each predicted PQS. The viral genome length is reported above the density plot.

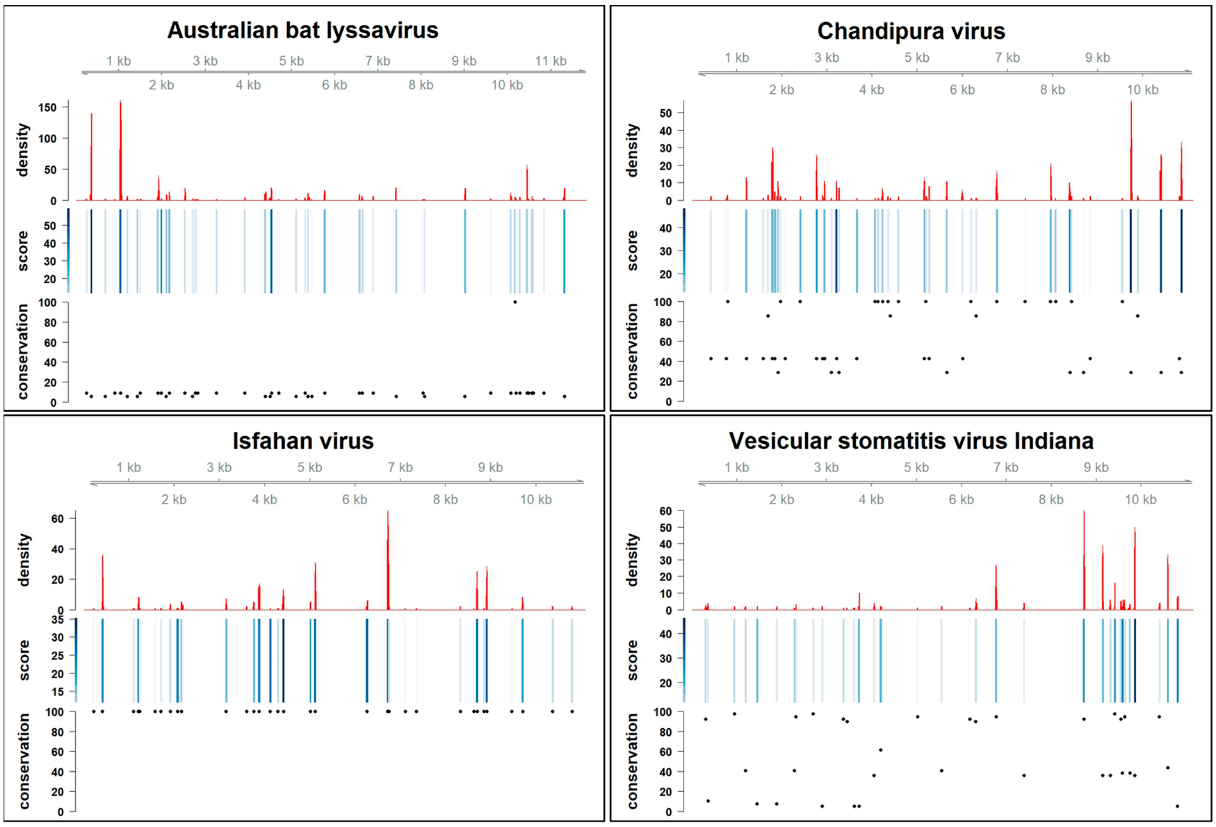

Figure S3. Presence, density, score and conservation of PQSs in negative-strand linear ssRNA genome viruses

Plots representing the PQS density (red bars), the score (blue bars) and the conservation percentage (black dots) of each predicted PQS. The viral genome length is reported above the density plot.

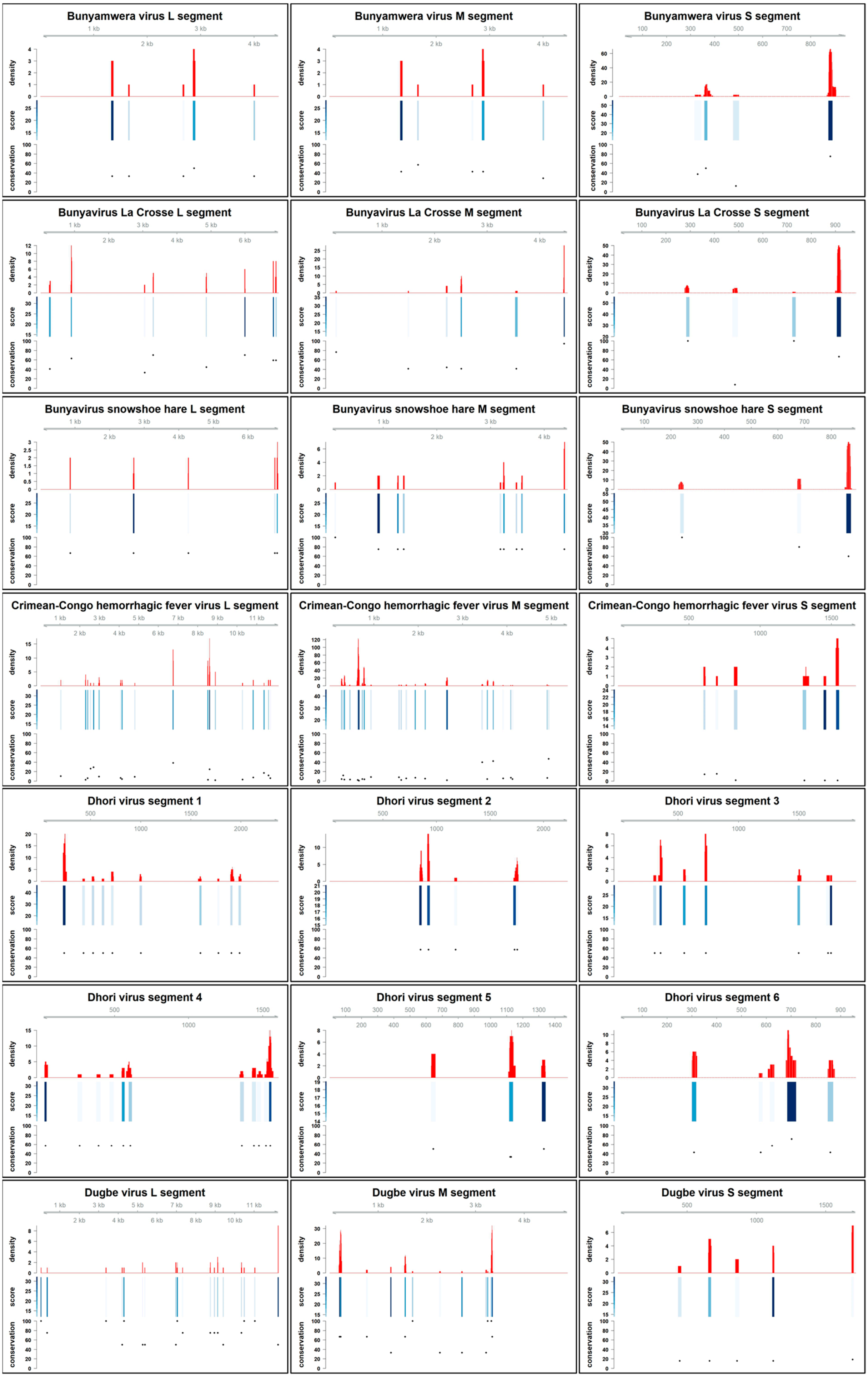

Bunyamwera virus L segment
Bunyamwera virus M segment
Bunyamwera virus S segment
Bunyavirus La Crosse L segment
Bunyavirus La Crosse M segment
Bunyavirus La Crosse S segment
Bunyavirus snowshoe hare L segment
Bunyavirus snowshoe hare M segment
Bunyavirus snowshoe hare S segment
Crimean-Congo hemorrhagic fever virus L segment
Crimean-Congo hemorrhagic fever virus M segment
Crimean-Congo hemorrhagic fever virus S segment
Dhori virus segment 1
Dhori virus segment 2
Dhori virus segment 3
Dhori virus segment 4
Dhori virus segment 5
Dhori virus segment 6
Dugbe virus L segment
Dugbe virus M segment
Dugbe virus S segment
density
score
conservation

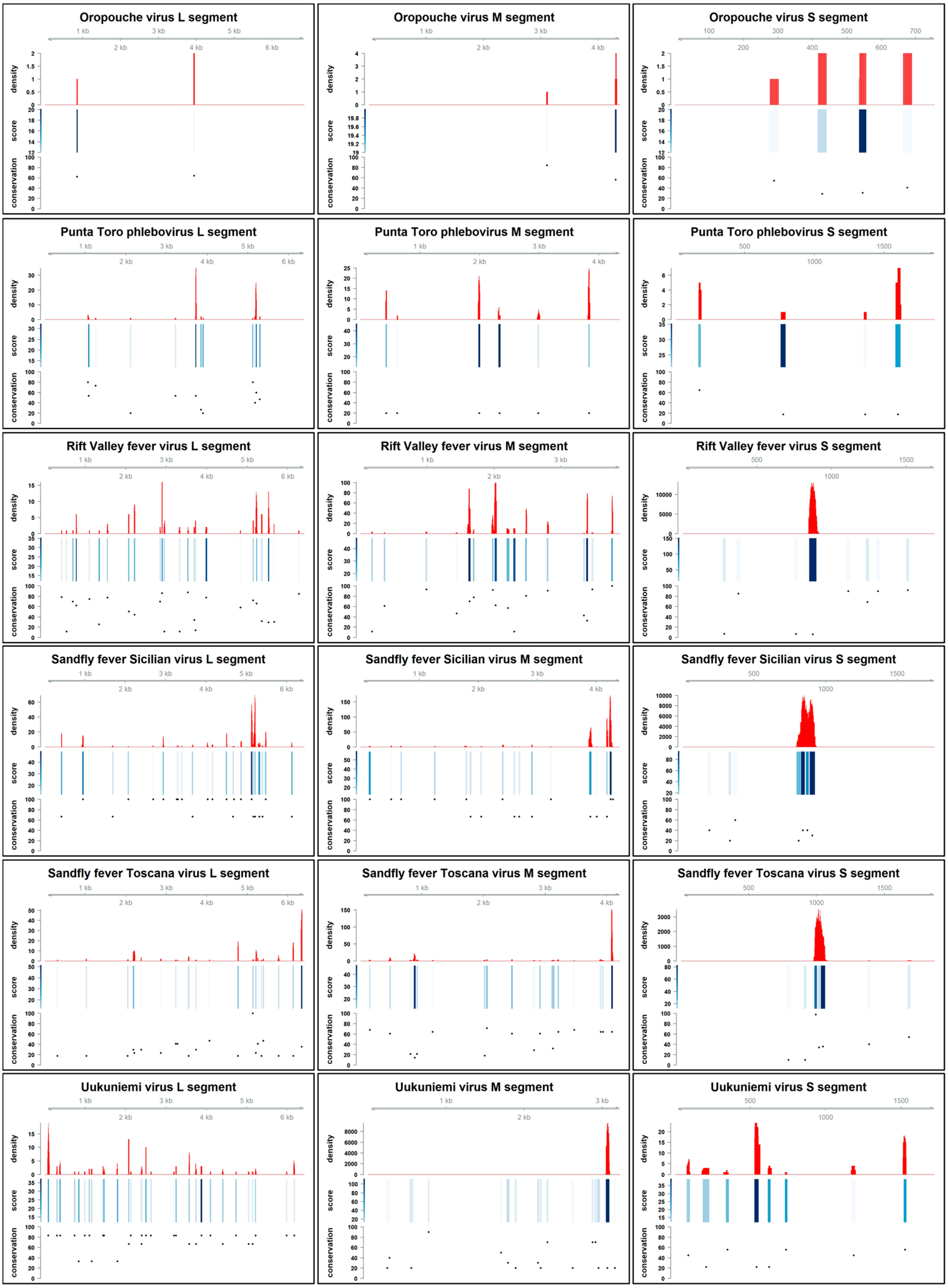

Figure S4. Presence, density, score and conservation of PQSs in negative-strand segmented ssRNA genome viruses

Plots representing the PQS density (red bars), the score (blue bars) and the conservation percentage (black dots) of each predicted PQS. The viral genome length is reported above the density plot. The viral segments are in alphabetical orders. In panels with long virus names, the word segment has been abbreviated to "seg".

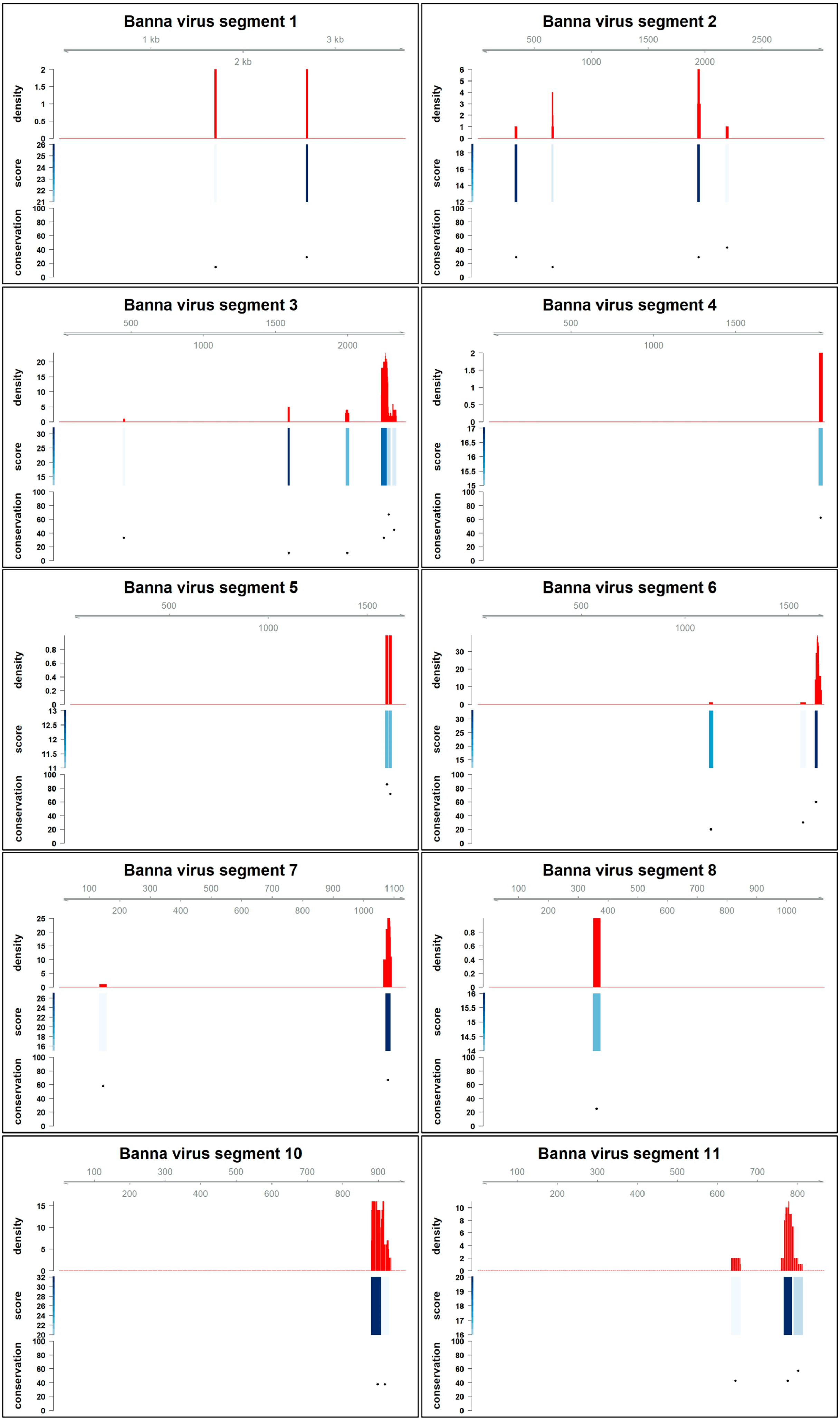
Banna virus segment 1
1 kb
3 kb
2 kb
density
score
conservation
Banna virus segment 2
500
1500
2500
1000
2000
density
score
conservation
Banna virus segment 3
500
1500
1000
2000
density
score
conservation
Banna virus segment 4
500
1500
1000
density
score
conservation
Banna virus segment 5
500
1500
1000
density
score
conservation
Banna virus segment 6
500
1500
1000
density
score
conservation
Banna virus segment 7
100
300
500
700
900
1100
200
400
600
800
1000
density
score
conservation
Banna virus segment 8
100
300
500
700
900
200
400
600
800
1000
density
score
conservation
Banna virus segment 10
100
300
500
700
900
200
400
600
800
density
score
conservation
Banna virus segment 11
100
300
500
700
200
400
600
800
density
score
conservation

Figure S5. Presence, density, score and conservation of PQSs in dsRNA genome viruses

Plots representing the PQS density (red bars), the score (blue bars) and the conservation percentage (black dots) of each predicted PQS. The viral genome length is reported above the density plot. Segment 9 contains no PQS and it is not represented.

Positive polarity ssRNA Flaviviruses

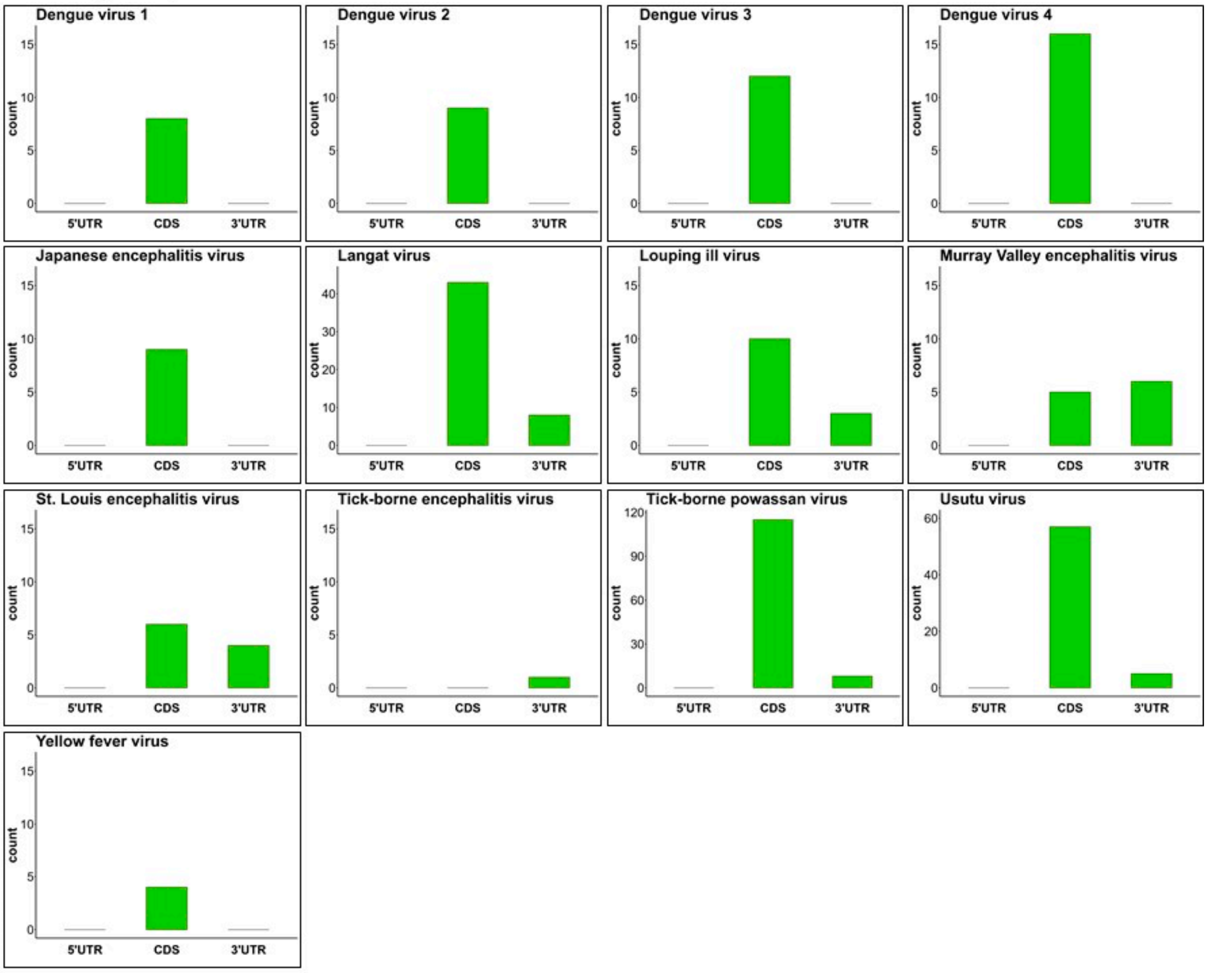

Positive polarity ssRNA Alphaviruses

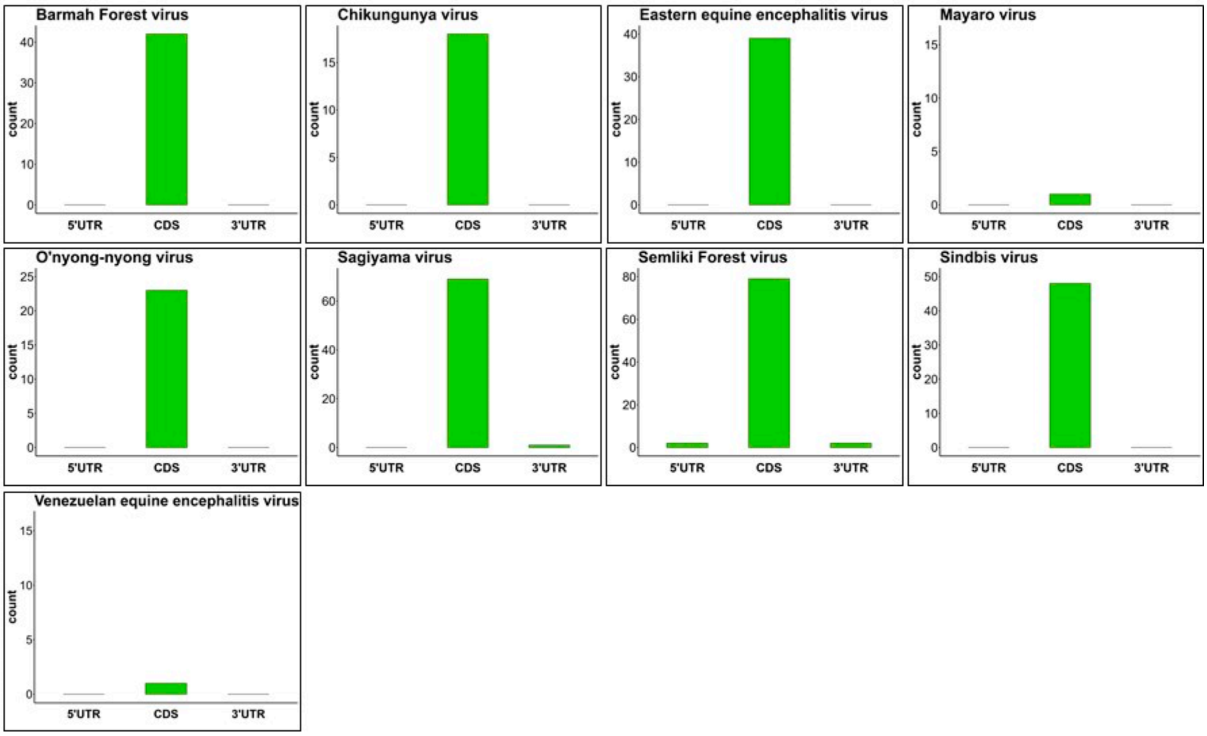

Segmented negative polarity ssRNA viruses

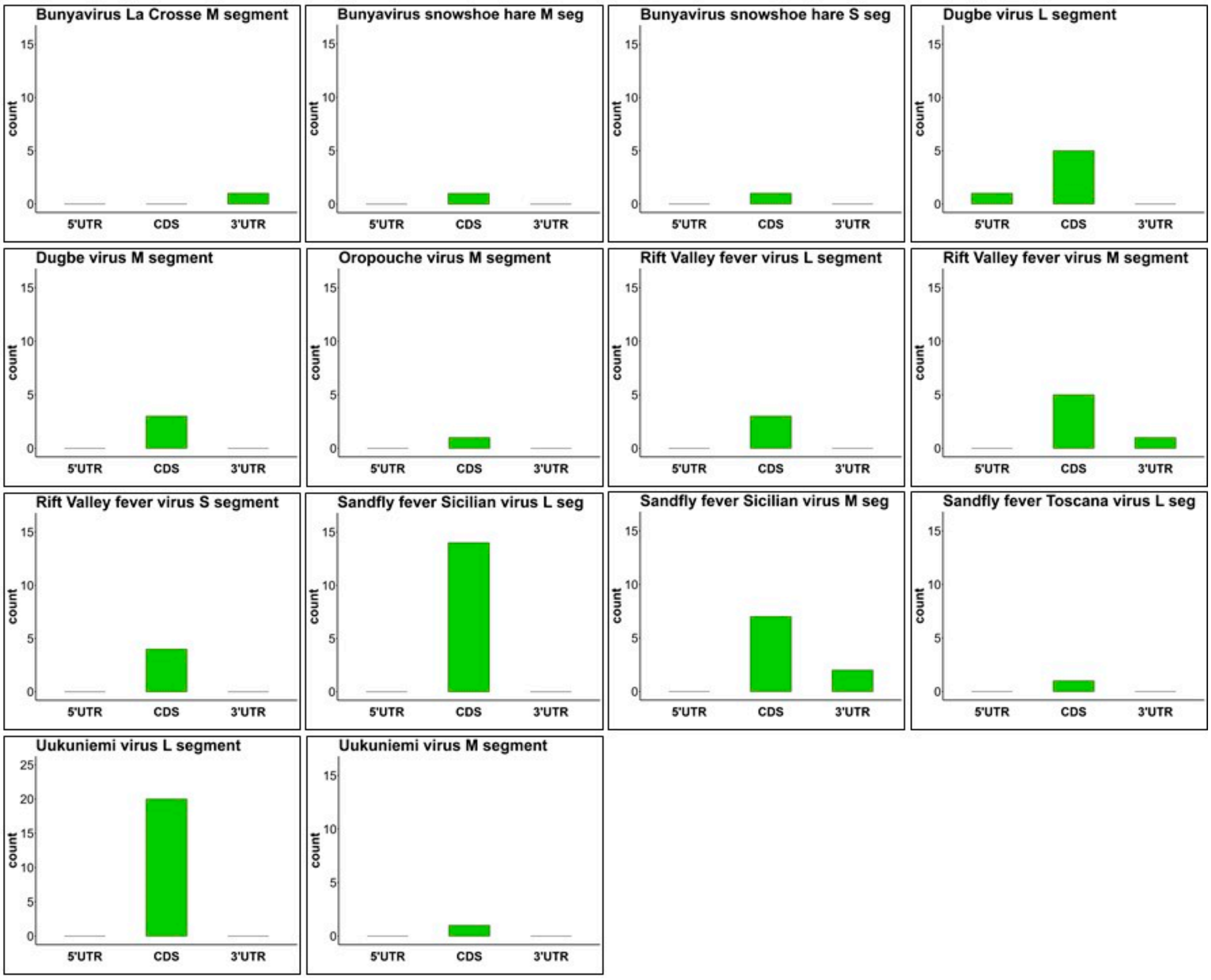

Single linear negative polarity ssRNA

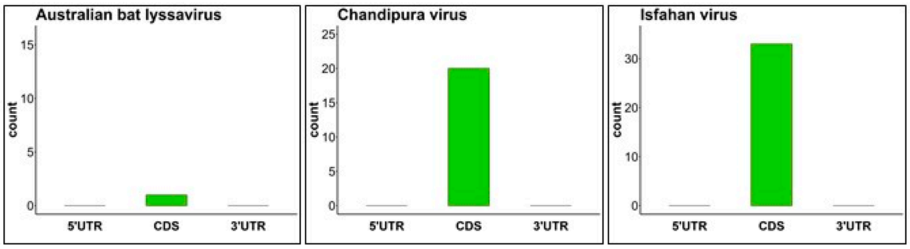

Figure S6. Conserved PQSs genomic localization in arboviruses

Plots reporting the annotation of highly conserved PQSs. Each viral genome was divided into three regions: Untranslated regions (5′ and 3′ UTRs) and coding sequences (CDS). PQSs were annotated on the basis of the official NCBI annotation of each viral Reference genome. In panels with long virus names, the word segment has been abbreviated to "seg".

Additional supplementary material provided: Zipped file containing the complete set of aligned viral genomes in FASTA format (aligned_genomes.zip).
